# Atherosclerosis after pre‐eclampsia: systematic review and meta‐analysis

**DOI:** 10.1002/uog.70014

**Published:** 2025-08-31

**Authors:** G. Jansen, A. de Rooy, E. Janssen, S. Altintas, A. van 't Hof, C. Mihl, B. Kietselaer, M. Spaanderman, C. Ghossein‐Doha

**Affiliations:** ^1^ GROW School for Oncology and Reproduction Maastricht University Maastricht The Netherlands; ^2^ Department of Obstetrics and Gynaecology Maastricht University Medical Centre+ (MUMC+) Maastricht The Netherlands; ^3^ Department of Cardiology Zuyderland Medical Centre Heerlen The Netherlands; ^4^ Faculty of Health, Medicine and Life Sciences Maastricht University Maastricht The Netherlands; ^5^ Cardiovascular Research Institute Maastricht (CARIM) Maastricht University Maastricht The Netherlands; ^6^ Faculty of Health and Life Sciences University of Hasselt Hasselt Belgium; ^7^ Department of Cardiology Maastricht University Medical Centre+ (MUMC+) Maastricht The Netherlands; ^8^ Department of Radiology and Nuclear Medicine Maastricht University Medical Centre+ (MUMC+) Maastricht The Netherlands; ^9^ Department of Cardiology, Mayo Clinic Rochester NY USA; ^10^ Department of Obstetrics and Gynaecology Radboud University Medical Centre+ Nijmegen The Netherlands; ^11^ Department of Cardiology Erasmus Medical Centre Rotterdam The Netherlands

**Keywords:** atherosclerosis, cardiovascular disease, non‐invasive imaging, plaque, pre‐eclampsia, premature aging

## Abstract

**Objective:**

Pre‐eclampsia complicates up to 8% of pregnancies and is associated with increased risk of ischemic cardiac and cerebral disease, which may be prevented through management of cardiovascular risk when early disease stages are detected. This meta‐analysis aimed to determine the prevalence of clinical and subclinical atherosclerosis in women after pre‐eclamptic *vs* non‐pre‐eclamptic pregnancy with advancing maternal age.

**Methods:**

A systematic search of the literature was conducted in PubMed, Embase and Web of Science for studies reporting on the prevalence of atherosclerosis in women with a previous pre‐eclamptic pregnancy and those with a previous uncomplicated pregnancy. Any systemic atherosclerosis documented using ultrasound or computed tomography was included. Random‐effects meta‐analysis was used to compute the odds ratio (OR) with 95% CI for the association between pre‐eclampsia and the presence of atherosclerosis. Subgroup analysis was conducted according to average maternal age at evaluation.

**Results:**

A total of 11 articles were included (13 217 participants). The average maternal age at evaluation ranged from 32 to 60 years. Within this age range, the pooled OR for the presence of atherosclerotic plaque after pre‐eclampsia was 1.57 (95% CI, 1.39–1.78). The pooled OR of developing atherosclerotic plaque after a pre‐eclamptic *vs* non‐pre‐eclamptic pregnancy increased gradually with advancing maternal age. The OR was not significant in the 30–39‐year‐old group (0.64 (95% CI, 0.10–4.15)), but the odds of finding an atherosclerotic plaque were significantly increased after pre‐eclamptic pregnancy in the 40–49‐year‐old group (OR, 1.59 (95% CI, 1.34–1.89)) and 50–60‐year‐old group (OR, 2.00 (95% CI, 1.30–3.08)). At any given age, the percentage plaque prevalence in formerly pre‐eclamptic women was roughly equal to that seen 10 years later in women with a previous non‐pre‐eclamptic pregnancy.

**Conclusions:**

Women with a previous pre‐eclamptic pregnancy exhibit atherosclerosis more frequently and approximately 10 years earlier compared with women with a previous non‐pre‐eclamptic pregnancy. Targeted primary prevention is required to reduce morbidity and mortality from premature cardiovascular disease in women after pre‐eclampsia. © 2025 The Author(s). *Ultrasound in Obstetrics & Gynecology* published by John Wiley & Sons Ltd on behalf of International Society of Ultrasound in Obstetrics and Gynecology.

## INTRODUCTION

In recent years, there has been increased awareness that cardiovascular disease (CVD) is a leading cause of female mortality, accounting for one‐third of all female deaths globally^1^. Despite increased recognition, CVD in women is still underdiagnosed and undertreated, ultimately leading to poorer overall health outcomes^2^, ^3^. Therefore, there is a compelling argument to focus on reducing the burden of CVD in women.

Typically, atherosclerosis develops slowly over many decades^4^. Alongside traditional risk factors, such as diabetes, hypertension and hypercholesterolemia, there are several female‐specific risk factors associated with the development of atherosclerosis and CVD. These include menopause, gestational diabetes, preterm birth and hypertensive disorders of pregnancy, including pre‐eclampsia^1^, ^5^.

Pre‐eclampsia is characterized by global endothelial dysfunction, as well as high blood pressure with systemic features^6^, which may lead to earlier development of atherosclerosis and ischemic cardiac and cerebral events^7^, ^8^. Pre‐eclampsia occurs in 2–8% of pregnancies and leads to a 2–8‐fold increased risk of future CVD^9^, ^10^. Pre‐eclampsia itself may lead to CVD^7^, ^8^; however, it is also possible that pregnancy is a ‘stress test’, where the development of pre‐eclampsia simply reveals an underlying predisposition to CVD^11^, ^12^. Meta‐analyses have shown that cardiovascular events, such as myocardial infarction and cerebrovascular accident, occur significantly more often after a pre‐eclamptic pregnancy^10^, ^13^. This suggests that there may be more underlying atherosclerosis in women after a pre‐eclamptic pregnancy compared with a non‐pre‐eclamptic pregnancy. Although a significant number of women have a raised CVD risk after pre‐eclampsia, only a subgroup of women will develop CVD requiring early primary intervention. Therefore, easily measurable and relevant intermediate risk factors are required.

To the best of our knowledge, no meta‐analysis has investigated directly the presence of atherosclerotic plaques nor the development of accelerated atherosclerosis after pre‐eclamptic pregnancy. The objectives of this meta‐analysis were to determine the prevalence of atherosclerotic plaque in any systemic maternal blood vessel after a pre‐eclamptic *vs* non‐pre‐eclamptic pregnancy and to investigate the rate of development of atherosclerosis by undertaking sub‐analyses based on maternal age. Our hypothesis was that there would be a higher prevalence of atherosclerosis after pre‐eclamptic pregnancy. This investigation could help to inform risk assessment and CVD prevention in women after a pregnancy affected by pre‐eclampsia.

## METHODS

The study protocol was registered in the PROSPERO online database of systematic reviews (reference number CRD42022289635). The study was conducted and reported in accordance with the PRISMA 2020^14^ and MOOSE^15^ checklists.

### Inclusion/exclusion criteria

All articles published until 22 November 2023 were considered for inclusion. No restriction was placed on the definition of atherosclerosis used. Clinical and subclinical definitions of atherosclerosis were accepted. Furthermore, any definition of pre‐eclampsia and HELLP (Hemolysis, Elevated Liver enzymes and Low Platelets) syndrome was accepted. Uncomplicated pregnancies were defined as those without pre‐eclampsia or HELLP syndrome.

Studies were limited to those that included women who had pre‐eclampsia or HELLP syndrome in a previous pregnancy, in addition to control women who had a previous uncomplicated pregnancy. In addition, eligible studies had to carry out postpartum imaging of maternal vessels using any type of computed tomography (CT) or ultrasound imaging to determine the presence of atherosclerotic plaque. Follow‐up had to start after the initial 6‐week postpartum period and could extend indefinitely.

Only primary quantitative research was included, from which it was possible to extract data required for analysis. Articles were limited to those available in Dutch or English (including available translations) and for which the full text was available.

### Search strategy and study selection

PubMed, Embase and Web of Science databases were searched for relevant studies. The detailed search strategy is provided in Appendix S1. Additionally, reference lists of the included studies, other meta‐analyses and review articles were screened for potentially eligible studies.

After removal of duplicates, two reviewers (G.J., A.D.R.) independently screened all titles and abstracts, and subsequently full‐text articles, using the inclusion/exclusion criteria. Disagreements were resolved by consensus, and a third reviewer (C.G.‐D.) was involved if necessary.

### Data extraction and risk‐of‐bias assessment

Data were extracted from included studies by two reviewers (G.J., A.D.R.) using a standardized template, including publication date, study design, country, sample size, imaging modality used, artery visualized, previous complicated or uncomplicated pregnancy, pre‐eclampsia definition used, population characteristics, duration of follow‐up, timing of assessment and prevalence of atherosclerotic plaque. If the available published data were insufficient, we attempted to obtain this information directly from the authors. Missing data are indicated where applicable. Women with a previous pre‐eclamptic pregnancy were defined as cases and women with a previous non‐pre‐eclamptic pregnancy were defined as controls. Studies were classified into subgroups based on imaging modality used/artery visualized and maternal age at evaluation.

The quality of included articles was assessed by a single reviewer (G.J.) using the Newcastle–Ottawa scale. This evaluated the domains of selection, comparability and outcome/exposure. The score for each article was converted to give an overall rating according to the Agency for Healthcare Research and Quality standards. Studies were classified as poor (selection, 0–1 star; comparability, 0 stars; and outcome/exposure, 0–1 star), fair (selection, 2 stars; comparability, 1–2 stars; and outcome/exposure, 2–3 stars) or good (selection, 3–4 stars; comparability, 1–2 stars; and outcome/exposure, 2–3 stars)^16^.

### Data synthesis and analysis

SPSS version 29.0 (IBM Corp., Armonk, NY, USA) was used to conduct the statistical analysis. Effect size was quantified using odds ratios (ORs) with 95% CIs for each study using available data on plaque prevalence. ORs from different studies were pooled using a random‐effects meta‐analysis. Forest plots were used to visualize the distribution of these data. We conducted subgroup analyses according to imaging modality used/artery visualized and average maternal age at evaluation, using a random‐effects meta‐analysis. Meta‐regression was used to analyze the influence of maternal age on plaque prevalence for both cases and controls.

The effect of maternal age on plaque prevalence was investigated visually using a scatter plot. Linear regression was used to determine trendlines for plaque prevalence when comparing cases and controls. The difference in trendlines between groups was determined using the general linear model.

Heterogeneity was assessed using the *I* ^2^ statistic, which quantifies the proportion of total variation due to differences between the included studies rather than sampling error. The *I* ^2^ statistic ranges from 0 to 100%, whereby 0% indicates no heterogeneity (observed variation is due to chance) and 100% indicates high heterogeneity (extreme variability where other factors are likely to be influencing the results)^17^. It was interpreted according to Cochrane guidelines as follows: 0–40% indicated insignificant heterogeneity, 30–60% indicated moderate heterogeneity, 50–90% indicated substantial heterogeneity and 75–100% indicated considerable heterogeneity^18^. Publication bias was assessed using Egger's test and visualized using a funnel plot.

Sensitivity analyses were performed in which we included only studies that reported adjusted ORs (aOR) to account for the influence of confounders. This included random‐effects meta‐analysis for the overall and subgroup analyses, as well as meta‐regression for maternal age.

## RESULTS

### Study selection

**Figure 1:**
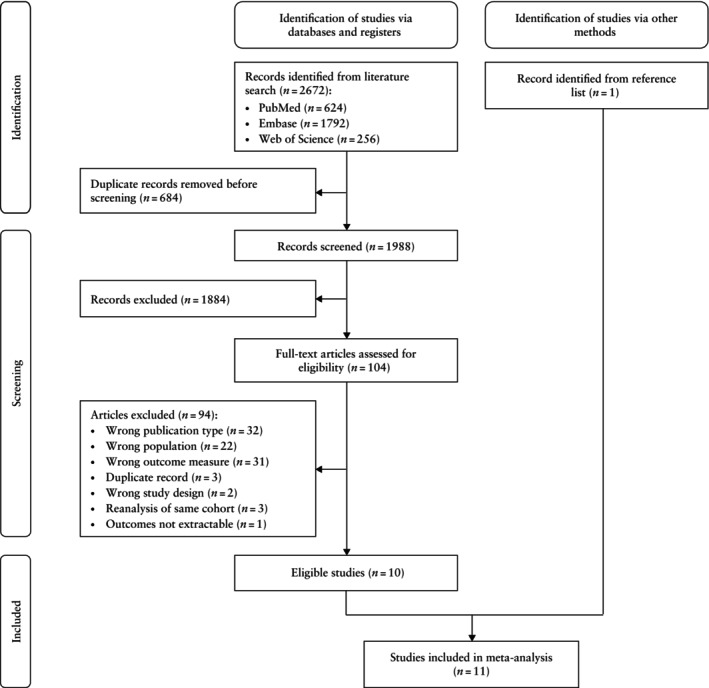
PRISMA flowchart summarizing inclusion of studies on the prevalence of atherosclerosis in women with previous pre‐eclamptic pregnancy and those with previous uncomplicated pregnancy in meta‐analysis.

A total of 2672 records were identified by the literature search, of which 684 were duplicates that were removed before title/abstract screening (Figure 1). A total of 1988 abstracts were screened, resulting in 1884 exclusions, primarily because of the wrong population or wrong outcome measure. The 104 remaining records underwent full‐text assessment for eligibility. There were a further 94 exclusions, primarily due to wrong publication type (*n* = 32), wrong population (*n* = 22) or wrong outcome measure (*n* = 31) (Table S1). The 10 remaining studies were included, in addition to a study identified manually from the reference lists. Thus, 11 studies^19^, ^20^, ^21^, ^22^, ^23^, ^24^, ^25^, ^26^, ^27^, ^28^, ^29^ were included in the meta‐analysis.

### Study characteristics

Across the 11 included studies^19^, ^20^, ^21^, ^22^, ^23^, ^24^, ^25^, ^26^, ^27^, ^28^, ^29^, there were a total of 13 217 participants (1959 cases and 11 258 controls) (Table 1). The study design was either cohort (*n* = 6)^19^, ^22^, ^23^, ^24^, ^26^, ^28^ or cross‐sectional (*n* = 5)^20^, ^21^, ^25^, ^27^, ^29^. Ten studies were carried out in Western countries^20^, ^21^, ^22^, ^23^, ^24^, ^25^, ^26^, ^27^, ^28^, ^29^, including two studies from each of Canada^21^, ^26^, Sweden^25^, ^27^, Denmark^23^, ^24^ and the USA^28^, ^29^. Iraq was the only non‐Western country included^19^. Ethnicity was often not specified; however, one study included only women with Northern European ancestry^22^ and another studied only African American women^29^. The majority of studies (*n* = 6) were published after 2019^19^, ^20^, ^21^, ^22^, ^24^, ^27^.

**Table 1:** Characteristics of 11 studies on the prevalence of atherosclerosis in women with previous pre‐eclamptic pregnancy and those with previous uncomplicated pregnancy included in meta‐analysis, according to average maternal age at evaluation (MA)

MA group/ study	Country	Study design	Imaging modality	Artery visualized; details	PE definition	Cases (*n*)	Controls (*n*)	Control pregnancy	Matched variables (ratio)	MA (years); years postpartum*		Overall MA (years)†	Weighting (%)
										Cases	Controls		
*30–39 years*													
Barr(2022)^21^	Canada	Prosp cross‐sectional	US	Carotid; bilateral; bulb	ACOG (2020)^73^	30	30	Uncomplicated	None	32.0 ± 3.9; 1.8 ± 1.4‡	34.3 ± 3.6; 2.3 ± 1.3‡	33.2	0.43
*40–49 years*													
Amor(2021)^20^	Spain	Prosp cross‐sectional	US	Carotid; bilateral; bulb, internal, common	ISSHP (2001)^74^	28	28	No PE history	MA ± 3 years, BMI, smoking, PPI	45.5 ± 7.1; 9.8†	44.2 ± 8.7; 9.8†	45.3	0.54
Benschop(2020)^22^	The Netherlands	Prosp cohort; northern European ancestry only	Non‐contrast CT	Coronary	ISSHP (2014)^75^	258	644	Normotensive	MA, ethnicity	46.0 [41.1–54.5]; 16.3 ± 5.9¶	46.0 [40.2–56.0]; 20.0 ± 8.2¶	46.0	11.05
Christensen(2016)^23^	Denmark	Prosp cohort	US	Carotid; bilateral; entire length	New‐onset HTN (SBP ≥ 140 mmHg and/or DBP ≥ 90 mmHg) and proteinuria (≥ 300 mg/24 h or ≥ 30 mg/mmol ACR (random urine) or ≥ 1 + on repeat dipstick)	20	20	Normotensive	MA, PPI	40.7 ± 2.7; 10.3 ± 0.7§	40.7 ± 2.3 10.3 ± 0.5	40.7	0.24
Hauge(2022)^24^	Denmark	Prosp cohort	Contrast and non‐contrast CT	Coronary; 17 segments	ICD‐10 codes^76^: O14.0, O14.1, O14.2, O14.9, O15.0, O15.9, Z358Q	704	706	No PE history	MA at CT, parity (1:1)	46.8 ± 4.4; 14.5 ± 6.1§	47.2 ± 4.3; 18.8¶	47.0	24.46
McDonald(2013)^26^	Canada	Prosp cohort	US	Carotid; bilateral; bifurcation, proximal internal, common	NHBPEP (1990)^77^	109	219	No PE history	MA ± 3 years, child age ± 5 years (1:2)	49.0 (44.0–55.0); 19.0 (15.0–25.0)¶	49.0 (45.0–56.0); 21.0 (16.0–28.0)¶	49.0	6.09
Wichmann(2019)^29^	USA	Retro cross‐sectional; African American women only	Contrast and non‐contrast CT	Coronary	NR	137	445	Uncomplicated	MA at CT, MA at first delivery, gravidity, BMI, smoking (1:1)	43.9†; 21.9§	44.0†; 23.0	44.0	8.29
*50–60 years*													
Al‐Gburi(2022)^19^	Iraq	Prosp cohort	Non‐contrast CT	Coronary	Validated PE questionnaire^78^	100	100	Normotensive	None	49.3; NR	49.7; NR	49.5	2.44
Haukkamaa(2009)^25^	Sweden	Prosp cross‐sectional	US	Carotid; right; bulb	ICD‐7 codes^79^: 642; ICD‐8 codes^80^: 637, 643.10, 644.10; ICD‐9 codes^81^: 6424–6427, 6461A, 6462A; ICD‐10 codes^76^: O14, O15	35	489	Uncomplicated	None	57.2 ± 6.5; NR	57.2 ± 8.4; NR	57.2	2.47
Lawesson(2023)^27^	Sweden	Prosp cross‐sectional	Contrast and non‐contrast CT	Coronary; 18 segments	ICD‐8 codes^80^: 637.03, 637.04, 637.09, 637.10, 637.99; ICD‐9 codes^81^: 642E, 642F, 642G, 642H; ICD‐10 codes^76^: O11, O14, O15	499	8537	Uncomplicated	None	56.8 (53.1–60.7); 29.6†	57.2 (53.6–61.1); 29.6†	56.8	42.32
White(2016)^28^	USA	Prosp cohort	Non‐contrast CT	Coronary	SBP > 140 mmHg or DBP > 90 mmHg on ≥ 2 occasions ≥ 4 h apart > 20 weeks’ gestation and new‐onset proteinuria (urine dipstick 1 +, proteinuria ≥ 0.300 g/24 h or PCR ≥ 0.3 g/24 h)	39	40	Normotensive	MA at first delivery, parity	59.4 ± 4.8; 34.9 (32.9–36.7)¶	59.7 ± 4.5; 34.5 (33.6–36.7)¶	59.5	1.66

Only first author is given for each study. Complete definitions of pre‐eclampsia (PE) are provided in Table S2. Weighting is for random‐effects meta‐analysis of any atherosclerotic plaque.

* Data are given as mean ± SD, mean, median [90% range] or median (interquartile range); years postpartum are from last pregnancy (‡), first complicated pregnancy (§), first pregnancy (¶) or unspecified (no symbol).

^†^ Mean was estimated using available data. ACOG, American College of Obstetricians and Gynecologists; ACR, albumin‐to‐creatine ratio; BMI, body mass index; CT, computed tomography; DBP, diastolic blood pressure; HTN, hypertension; ICD, International Statistical Classification of Diseases and Related Health Problems; ISSHP, International Society for the Study of Hypertension in Pregnancy; NHBPEP, National High Blood Pressure Education Program; NR, not reported; PCR, protein‐to‐creatine ratio; PPI, postpartum interval; Prosp, prospective; Retro, retrospective; SBP, systolic blood pressure; US, ultrasound.

Various definitions were used for pre‐eclampsia (Tables 1 and S2). All studies included exclusively women with a history of pre‐eclampsia as cases, with the exception of Hauge *et al*.^24^, who also included participants with a history of isolated HELLP syndrome in their case group. For the control group, four studies included normotensive pregnancies^19^, ^22^, ^23^, ^28^, four included uncomplicated pregnancies^21^, ^25^, ^27^, ^29^ and three included women without any history of pre‐eclampsia^20^, ^24^, ^26^.

For each individual study, both postpartum interval and maternal age at evaluation were comparable between cases and controls (Table 1). The average postpartum interval ranged from 2 to 35 years, calculated from the last pregnancy, first pregnancy or first complicated pregnancy. Most studies had a postpartum interval of at least 10 years. The average maternal age at evaluation ranged from 32 to 60 years. Studies were grouped according to average maternal age at evaluation as follows: 30–39 years (one study, 60 women)^21^, 40–49 years (six studies, 3318 women)^20^, ^22^, ^23^, ^24^, ^26^, ^29^ and 50–60 years (four studies, 9839 women)^19^, ^25^, ^27^, ^28^.

Five studies (1008 women) used ultrasound to visualize the carotid artery^20^, ^21^, ^23^, ^25^, ^26^ and six studies (12 209 women) used CT to image the coronary arteries^19^, ^22^, ^24^, ^27^, ^28^, ^29^ (Table 1). No study was included that visualized maternal vessels other than the carotid and coronary artery. All studies employed clear definitions to classify plaque. All 11 studies evaluated calcified plaque by visual identification, quantification of coronary artery calcification (CAC) using the Agatston score or both^19^, ^20^, ^21^, ^22^, ^23^, ^24^, ^25^, ^26^, ^27^, ^28^, ^29^. Nine studies (5/5 ultrasound studies^20^, ^21^, ^23^, ^25^, ^26^ and 4/6 CT studies^22^, ^24^, ^27^, ^29^) also evaluated non‐calcified and mixed plaque.

### Quality assessment

The quality of the 11 included studies^19^, ^20^, ^21^, ^22^, ^23^, ^24^, ^25^, ^26^, ^27^, ^28^, ^29^ was assessed using the Newcastle–Ottawa scale^16^ (Table S3). Most studies were rated as either fair (*n* = 4)^19^, ^22^, ^24^, ^26^ or good (*n* = 6)^20^, ^23^, ^25^, ^27^, ^28^, ^29^. The sole study in the 30–39‐year‐old group was classified as poor quality^21^. Given that this was the only study in this age group, it was included in the analysis. None of the studies were able to demonstrate that plaque formation was not present before the pre‐eclamptic or non‐pre‐eclamptic pregnancy. Likewise, none of the studies could demonstrate adequacy of cohort follow‐up, as this information was not available.

### Heterogeneity

For all meta‐analyses performed, including the sensitivity analyses, the *I* ^2^ statistic ranged from 0 to 39%, indicating insignificant heterogeneity. This suggests that the observed variation was likely due to chance. The only exception was the *I* ^2^ value of 49% for the prevalence of any plaque in the 50–60‐year‐old group, which signified moderate heterogeneity (Table 2).

**Table 2:** Odds ratios (OR) for presence of any atherosclerotic plaque after pre‐eclamptic pregnancy (cases) *vs* non‐pre‐eclamptic pregnancy (controls), according to imaging modality used/artery visualized and maternal age at evaluation

Maternal age group	Ultrasound/carotid artery					CT/coronary artery					Any imaging modality/any artery				
	Studies (*n* ^ref^)	Plaque prevalence (*n*/*N* (%))		OR (95% CI)	*I* ^2^ (%)	Studies (n^ref^)	Plaque prevalence (*n*/*N* (%))		OR (95% CI)	*I* ^2^ (%)	Studies (*n* ^ref^)	Plaque prevalence (*n*/*N* (%))		OR (95% CI)	*I* ^2^ (%)
		Cases	Controls				Cases	Controls				Cases	Controls		
30–39 years	1^21^	2/30 (6.7)	3/30 (10.0)	0.64 (0.10–4.15)	—	0	—	—	—	—	1^21^	2/30 (6.7)	3/30 (10.0)	0.64 (0.10–4.15)	—
40–49 years	3^20^, ^23^, ^26^	48/157 (30.6)	58/267 (21.7)	1.81 (1.13–2.88)	0	3^22^, ^24^, ^29^	292/1099 (26.6)	335/1795 (18.7)	1.56 (1.29–1.87)	0	6^20^, ^22^, ^23^, ^24^, ^26^, ^29^	340/1256 (27.1)	393/2062 (19.1)	1.59 (1.34–1.89)	0
50–60 years	1^25^	10/35 (28.6)	59/489 (12.1)	3.00 (1.37–6.53)	—	3^19^, ^27^, ^28^	222/638 (34.8)	2435/8677 (28.1)	1.77 (1.15–2.70)	39	4^19^, ^25^, ^27^, ^28^	232/673 (34.5)	2494/9166 (27.2)	2.00 (1.30–3.08)	49
Pooled overall result	5^20^, ^21^, ^23^, ^25^, ^26^	60/222 (27.0)	120/786 (15.3)	2.00 (1.28–3.11)	8	6^19^, ^22^, ^24^, ^27^, ^28^, ^29^	514/1737 (29.6)	2770/10 472 (26.5)	1.54 (1.35–1.75)	0	11^19^, ^20^, ^21^, ^22^, ^23^, ^24^, ^25^, ^26^, ^27^, ^28^, ^29^	574/1959 (29.3)	2890/11 258 (25.7)	1.57 (1.39–1.78)	0

CT, computed tomography; ref, reference.

### Synthesis of results

#### Overall

**Figure 2:**
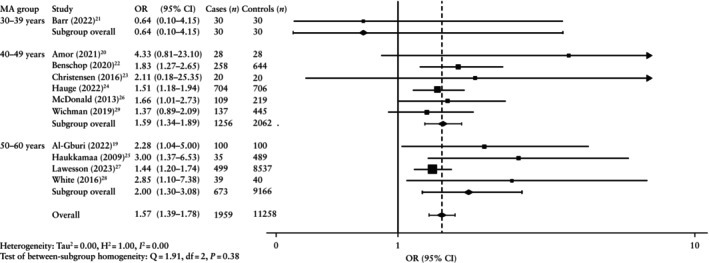
Forest plot showing random‐effects meta‐analysis for presence of any atherosclerotic plaque after pre‐eclamptic pregnancy (cases) *vs* non‐pre‐eclamptic pregnancy (controls), stratified by average maternal age at evaluation (MA). Only first author is shown for each study. Axis uses log scale. 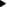 indicates cropping of upper bound of 95% CI. OR, odds ratio.

The overall pooled prevalence of plaque was higher after pre‐eclamptic compared with non‐pre‐eclamptic pregnancy (29.3% *vs* 25.7%) (Tables 2 and S4). Consequently, the pooled odds of developing atherosclerotic plaque were significantly higher after pre‐eclamptic compared with non‐pre‐eclamptic pregnancy (OR, 1.57 (95% CI, 1.39–1.78); *n* = 13 217) (Figure 2). Sensitivity analysis including only those studies that adjusted for the influence of confounders showed a similar aOR (1.37 (95% CI, 1.17–1.60); *n* = 11 249) (Figures S1 and S2).

#### Maternal age at evaluation

**Figure 3:**
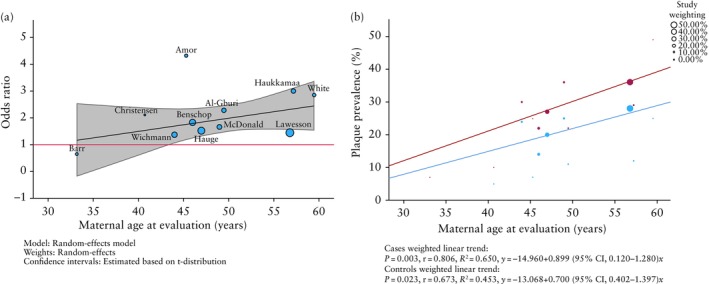
(a) Meta‐regression bubble plot showing odds ratio for presence of atherosclerotic plaque after pre‐eclamptic *vs* non‐pre‐eclamptic pregnancy, moderated by average maternal age at evaluation. Black solid line is meta‐regression prediction line, shading indicates 95% CI and red line shows where odds ratio becomes significant. First author is shown for each study. (b) Scatter plot with weighted lines of best fit showing prevalence of atherosclerotic plaque and average maternal age at evaluation for pre‐eclamptic pregnancies (red) and non‐pre‐eclamptic pregnancies (blue) in each study.

The pooled OR of developing any atherosclerotic plaque after pre‐eclamptic *vs* non‐pre‐eclamptic pregnancy increased gradually with advancing maternal age. The OR was non‐significant in the 30–39‐year‐old group (0.64 (95% CI, 0.10–4.15); *n* = 60), but the odds of finding any atherosclerotic plaque were significantly increased after pre‐eclamptic pregnancy in the 40–49‐year‐old group (OR, 1.59 (95% CI, 1.34–1.89); *n* = 3318) and 50–60‐year‐old group (OR, 2.00 (95% CI, 1.30–3.08); *n* = 9839) (Figure 2, Table 2). The sensitivity analysis including only those studies that adjusted for confounders showed a significant result for the 40–49‐year‐old group (aOR, 1.34 (95% CI, 1.02–1.76); *n* = 1410) but not for the 50–60‐year‐old group (aOR, 1.67 (95% CI, 0.91–3.09); *n* = 9839) (Figure S1). Meta‐regression showed that the odds of finding atherosclerotic plaque following a pre‐eclamptic pregnancy were significantly higher compared with the odds following a non‐pre‐eclamptic pregnancy after a maternal age of 44 years (Figure 3a). Although the sensitivity analysis also showed a similar trend of increasing aOR with advancing maternal age, it was not possible to compare this result because there were no studies in the earliest age group (Figure S3).

The prevalence of any atherosclerotic plaque after pre‐eclamptic pregnancy increased with increasing maternal age (30–39 years, 6.7%; 40–49 years, 27.1%; and 50–60 years, 34.5%) (Table 2). A similar trend was observed after non‐pre‐eclamptic pregnancy (10.0%, 19.1% and 27.2%, respectively). Figure 3b shows that, at any given age, the percentage plaque prevalence in formerly pre‐eclamptic women is roughly equal to that seen in controls 10 years later. Using weighting by study size, the expected increase for plaque prevalence was 0.9% per year (equivalent to 4.5% per 5 years) in the formerly pre‐eclamptic group and 0.7% per year (equivalent to 3.5% per 5 years) in the non‐pre‐eclamptic group. The increase in prevalence of atherosclerotic plaque in the formerly pre‐eclamptic group compared with the non‐pre‐eclamptic group was not statistically different (*P* = 0.563).

#### Calcified and non‐calcified plaque

The pooled OR of developing non‐calcified atherosclerotic plaque after a pre‐eclamptic *vs* non‐pre‐eclamptic pregnancy was 1.65 (95% CI, 1.27–2.14; *n* = 10 774) (Table S4). The corresponding OR for calcified plaque was 1.52 (95% CI, 1.23–1.88; *n* = 11 955). In previously pre‐eclamptic women, the overall reported prevalence of any plaque was 29.3%, of which 28.1% was non‐calcified and 71.9% was calcified. After non‐pre‐eclamptic pregnancy, the overall prevalence of plaque was 25.7%, of which 18.5% was non‐calcified and 81.5% was calcified.

**Figure 4:**
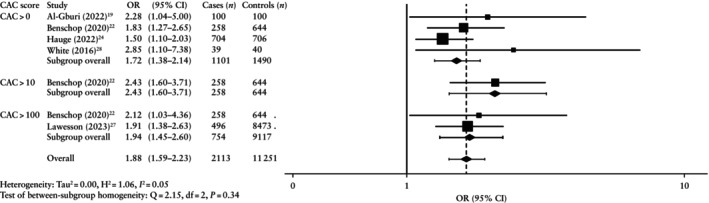
Forest plot showing random‐effects meta‐analysis for presence of calcified atherosclerotic plaque after pre‐eclamptic pregnancy (cases) *vs* non‐pre‐eclamptic pregnancy (controls), stratified by coronary artery calcification (CAC) score. Only first author is shown for each study. Axis uses log scale. OR, odds ratio.

Various CAC score cut‐offs were used by the included studies to classify calcified plaque (> 0, > 10 and > 100). For all CAC score categories, the prevalence of plaque was always higher in the formerly pre‐eclamptic group (Table S4, Figure 4).

#### Imaging modality/artery visualized

**Figure 5:**
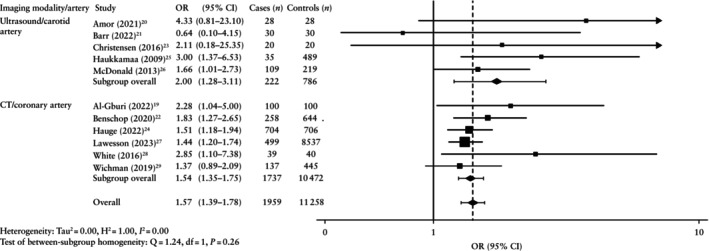
Forest plot showing random‐effects meta‐analysis for presence of any atherosclerotic plaque after pre‐eclamptic pregnancy (cases) *vs* non‐pre‐eclamptic pregnancy (controls), stratified by imaging modality used/artery visualized. Only first author is shown for each study. Axis uses log scale. 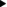 indicates cropping of upper bound of 95% CI. CT, computed tomography; OR, odds ratio.

Imaging with both ultrasound and CT indicated higher odds for observing atherosclerotic plaque formation after pre‐eclamptic pregnancy. The pooled overall OR was higher in the ultrasound/carotid artery group (2.00 (95% CI, 1.28–3.11); *n* = 1008) compared with the CT/coronary artery group (1.54 (95% CI, 1.35–1.75); *n* = 12 209), but the 95% CI overlapped between groups (Table 2, Figure 5). The sensitivity analysis adjusted for confounders showed the same trend (ultrasound: aOR, 3.62 (95% CI, 1.49–8.78), *n* = 524 and CT: aOR, 1.37 (95% CI, 1.13–1.56), *n* = 10 725) (Figure S2). For both imaging modalities, the OR for the presence of plaque increased with advancing maternal age at evaluation. Likewise, both ultrasound and CT showed a higher pooled prevalence of atherosclerotic plaque in cases compared with controls (27.0% *vs* 15.3% and 29.6% *vs* 26.5%, respectively).

### Publication bias

The symmetrical distribution of studies in the funnel plot (Figure S4) and the result of Egger's regression‐based test (*P* = 0.121) indicated non‐significant publication bias.

## DISCUSSION

### Main findings

Pre‐eclampsia predisposes women to early‐onset CVD. This meta‐analysis found a higher prevalence of atherosclerotic plaque in the three decades after pre‐eclamptic pregnancy compared with after non‐pre‐eclamptic pregnancy (OR, 1.57 (95% CI, 1.39–1.78)). This result excludes microvascular plaque, which is considered to be more prevalent in women, so is likely to underestimate the true atherosclerotic burden^30^. Furthermore, although plaque prevalence increased at a similar rate with advancing maternal age in both groups, the prevalence was always higher in formerly pre‐eclamptic women over the age range evaluated. At any given age, the prevalence of atherosclerotic plaque in formerly pre‐eclamptic women was roughly equivalent to that seen in control women 10 years later.

### Mechanisms and risk factors

Systemic endothelial dysfunction, hyperinflammation and hyperlipidemia during pre‐eclamptic pregnancy may cause *de‐novo* hypertension and hypercholesterolemia^31^, ^32^, ^33^, ^34^, ^35^, ^36^, ^37^, ^38^. It has been reported that, from 0.5 to 20 years after pre‐eclamptic pregnancy, hypertension was two to four times more prevalent and dyslipidemia was twice as prevalent compared with after uncomplicated pregnancy^39^. Hypertension causes shear stress and endothelial dysfunction^40^; meanwhile, hypercholesterolemia facilitates vascular cholesterol deposition, initiating and driving the progression of atherosclerotic plaque^41^. Although adjustment for confounders such as dyslipidemia and hypertension in the present meta‐analysis resulted generally in a reduction in the magnitude of the OR (Figures S1 and S2), these risk factors do not fully explain the increased plaque prevalence seen after pre‐eclampsia.

Persisting endothelial dysfunction may cause arterial stiffness and reduced vascular compliance^37^, ^38^. Loss of vascular compliance could initiate and advance plaque formation, especially when concurrent inflammation and oxidative stress are present^34^, ^35^, ^36^. These processes could explain the earlier appearance of atherosclerosis after pre‐eclampsia reported in this meta‐analysis^34^, ^42^, ^43^, ^44^. Alternatively, the same risk factors driving the emergence of pre‐eclampsia could initiate plaque formation before pregnancy, which progresses further after pre‐eclampsia^11^, ^12^, ^45^.

### Prognostic implications

This meta‐analysis included studies that reported either calcified plaque alone or composite measures of both non‐calcified and calcified plaque. Information on overall plaque burden or other features of plaque morphology was not available, despite these features having better predictive value for atherosclerotic CVD (ASCVD) events compared with plaque presence alone^46^, ^47^, ^48^.

CAC, reported using the Agatston score, indicates the presence of calcified plaque. A raised CAC score can predict an increased incidence of ASCVD events, including strokes and coronary events^49^, ^50^, ^51^. Compared with a CAC score of 0, a CAC score of > 0 is associated with an increase in ASCVD incidence of 2.92 per 1000 person‐years, whereas a CAC score of > 100 raises the incidence to 8.27 per 1000 person‐years^50^. Furthermore, the incidence of major adverse cardiac events may increase significantly with CAC > 200^52^.

Although a CAC score of 0 is associated with a very low risk of CVD events^50^, it does not exclude CVD risk in women after pre‐eclamptic pregnancy, as it fails to account for non‐calcified plaque. As a result, measures of calcification likely underestimate CVD risk. This meta‐analysis found that studies reporting composite measures of calcified and non‐calcified plaque documented higher plaque prevalence compared with those reporting solely calcified plaque (Table S4), and that roughly one‐third of composite plaque was non‐calcified in formerly pre‐eclamptic women. Risk estimates using CAC scores do not account for mixed and non‐calcified plaque, microvascular disease or non‐atherosclerotic cardiovascular events, even though these three conditions tend to occur more often in women compared with men^53^, ^54^, ^55^, ^56^, ^57^. There is evidence that non‐calcified plaque is associated with higher rates of CVD events in at‐risk populations, perhaps because of inherent plaque instability and vulnerability leading to plaque rupture^48^, ^58^, ^59^. Other features that increase plaque vulnerability and relate directly to ASCVD events include the ‘napkin ring sign’, positive remodeling, spotty calcification and low‐attenuation plaque^53^, ^60^, ^61^. To improve CVD risk prediction, imaging assessment should include these features in addition to measures of calcification.

### Recommendations and current guidelines

Current guidelines identify a history of pre‐eclampsia as a risk factor for ASCVD^62^, ^63^, and recommend that these women should undergo 5‐yearly risk‐factor screening for CVD^63^. The present meta‐analysis showed that one in five formerly pre‐eclamptic women at 40 years of age, and one in three at 50 years of age, had atherosclerotic plaque (calcified and non‐calcified). This suggests that CVD screening after pre‐eclampsia should start by 40 years of age, or perhaps earlier at 5‐years postpartum in women who gave birth before 35 years of age. Plaque prevalence in formerly pre‐eclamptic women at any given age was roughly equivalent to that seen in non‐pre‐eclamptic women 10 years later. Therefore, women with an uncomplicated pregnancy could undergo regular CVD screening when they reach the equivalent risk of atherosclerosis about 10 years after their formerly pre‐eclamptic counterparts, at age 50 years.

Current risk tools tend to underestimate ASCVD risk in young and formerly pre‐eclamptic women, complicating prevention in this at‐risk group^63^. Guidelines advise using CAC scoring when there is uncertainty regarding the initiation of preventive therapy for women with borderline or intermediate 10‐year ASCVD risk, and that CAC scoring can be considered for low‐risk patients^62^, ^63^. Given that one‐third of formerly pre‐eclamptic women with plaque had non‐calcified plaque, carotid ultrasonography or simultaneous identification of non‐calcified plaque using CT angiography with CAC scoring could be considered, in order to avoid underestimating risk in these women. Documented presence of plaque would reclassify these women as very high risk^62^. The lifetime risk of breast cancer, for a 100‐mSv radiation exposure, appears to be twice as high at 40 years of age (0.135% excess risk) compared with 50 years of age (0.067% excess risk)^64^. Given this and the high cost of CT, ultrasound may be the preferred imaging modality, especially for younger women^50^, ^64^, ^65^, ^66^, ^67^, ^68^.

Guidelines recommend assessing individual risk and considering both lifestyle intervention and pharmacological therapy, including statin therapy^62^, ^63^. Statins are a mainstay of CVD prevention: they reduce the CVD event rate by stabilizing plaques, and may induce plaque regression^69^, ^70^, ^71^. A Cochrane review reported a 5‐year number‐needed‐to‐treat for statins of 56 to prevent coronary heart disease events and 200 for stroke events^72^.

### Limitations

Our meta‐analysis has limited ethnic diversity and a relative lack of studies in women under 40 years of age, over 60 years of age and in the early postpartum period, limiting the generalizability of our findings. Due to shared risk factors for pre‐eclampsia and CVD, it would be ideal to determine plaque presence before pre‐eclampsia. However, none of the included studies could confirm the presence or absence of plaque before the pre‐eclamptic or uncomplicated pregnancy. Without measurements of pre‐existing plaque, the early maternal age group (30–39 years) could serve as a proxy for baseline plaque level, perhaps indicating no difference in the prevalence of pre‐existing plaque. Nonetheless, this singular small study, which scored poorly on the risk‐of‐bias assessment, cannot rule out a difference in baseline plaque level^21^. Moreover, differing definitions of atherosclerotic plaque between studies could have affected our results. Finally, some control groups may have included women with hypertensive disorders, possibly overestimating plaque prevalence in control pregnancies. Nonetheless, the difference between pre‐eclamptic and non‐pre‐eclamptic women would likely be greater in the absence of hypertensive controls.

### Conclusions

This meta‐analysis found that women with a history of pre‐eclampsia exhibit macrovascular atherosclerosis, on average, 10 years earlier compared with non‐pre‐eclamptic women. Formerly pre‐eclamptic women are at greater risk of ASCVD events and should undergo careful individual risk assessment. Considering a prevalence of atherosclerotic plaque of at least one in five after a pre‐eclamptic pregnancy from a maternal age of 40 years, carotid ultrasonography could be a safe tool to aid decision‐making, optimize lifestyle and pharmacological interventions, and reduce CVD morbidity and mortality.

## Supporting information


**Appendix S1** Search strategy.
**Table S1** Full‐text articles excluded and reason for exclusion.
**Table S2** Complete definitions of pre‐eclampsia used by included studies.
**Table S3** Quality assessment of included studies using Newcastle–Ottawa scale.
**Table S4** Odds ratios for presence of atherosclerotic plaque after pre‐eclamptic pregnancy (cases) *vs* non‐pre‐eclamptic pregnancy (controls), according to type of plaque and maternal age at evaluation.
**Figure S1** Forest plot showing sensitivity analysis for presence of any atherosclerotic plaque after pre‐eclamptic pregnancy (cases) *vs* non‐pre‐eclamptic pregnancy (controls), stratified by average maternal age at evaluation. Only studies in which odds ratios were adjusted for confounders are included.
**Figure S2** Forest plot showing sensitivity analysis for presence of any atherosclerotic plaque after pre‐eclamptic pregnancy (cases) *vs* non‐pre‐eclamptic pregnancy (controls), stratified by imaging modality used/artery visualized. Only studies in which odds ratios were adjusted for confounders are included.
**Figure S3** Meta‐regression bubble plot showing adjusted odds ratio for presence of atherosclerotic plaque after pre‐eclamptic *vs* non‐pre‐eclamptic pregnancy, moderated by average maternal age at evaluation. Only studies in which odds ratios were adjusted for confounders are included. Red line shows where odds ratio becomes significant.
**Figure S4** Funnel plot showing distribution of studies included in meta‐analysis according to odds ratio for presence of any atherosclerotic plaque after pre‐eclamptic *vs* non‐pre‐eclamptic pregnancy.

## Data Availability

The data that support the findings of this study are available from the corresponding author upon reasonable request.
