# Supplementary material for: Atherosclerosis after pre‐eclampsia: systematic review and meta‐analysis
Source: Ultrasound Obstet Gynecol. 2025 Aug 31;67(1):15–26. doi: 10.1002/uog.70014 (PMC12757827; doi:10.1002/uog.70014)
Supplement: Supplementary file 1 — Appendix S1 Search strategy. Table S1 Full‐text articles excluded and reason for exclusion. Table S2 Complete definitions of pre‐eclampsia used by included studies. Table S3 Quality assessment of included studies using Newcastle–Ottawa scale. Table S4 Odds ratios for presence of atherosclerotic plaque after pre‐eclamptic pregnancy (cases) vs non‐pre‐eclamptic pregnancy (controls), according to type of plaque and maternal age at evaluation. Figure S1 Forest plot showing sensitivity analysis for presence of any atherosclerotic plaque after pre‐eclamptic pregnancy (cases) vs non‐pre‐eclamptic pregnancy (controls), stratified by average maternal age at evaluation. Only studies in which odds ratios were adjusted for confounders are included. Figure S2 Forest plot showing sensitivity analysis for presence of any atherosclerotic plaque after pre‐eclamptic pregnancy (cases) vs non‐pre‐eclamptic pregnancy (controls), stratified by imaging modality used/artery visualized. Only studies in which odds ratios were adjusted for confounders are included. Figure S3 Meta‐regression bubble plot showing adjusted odds ratio for presence of atherosclerotic plaque after pre‐eclamptic vs non‐pre‐eclamptic pregnancy, moderated by average maternal age at evaluation. Only studies in which odds ratios were adjusted for confounders are included. Red line shows where odds ratio becomes significant. Figure S4 Funnel plot showing distribution of studies included in meta‐analysis according to odds ratio for presence of any atherosclerotic plaque after pre‐eclamptic vs non‐pre‐eclamptic pregnancy. [file UOG-67-15-s001.docx]

**Appendix S1** Search strategy

Pubmed

("pre eclampsia"[MeSH Terms] OR "pre eclamp*"[Title/Abstract] OR "pre-eclamp*"[Title/Abstract] OR "preeclamp*"[Title/Abstract] OR "Toxemia"[Title/Abstract] OR "Toxaemia"[Title/Abstract] OR "eclamp*"[Title/Abstract] OR "HELLP"[Title/Abstract] OR "PE"[Title/Abstract] OR "hypertension, pregnancy induced"[MeSH Terms] OR “maternal hypertension”[Title/Abstract])

AND

("ultrasonography, doppler"[MeSH Terms] OR "Echocardiography"[MeSH Terms] OR "computed tomography angiography"[MeSH Terms] OR "Angiography"[MeSH Terms] OR "diagnostic imaging"[MeSH Subheading] OR "Ultrasound"[Title/Abstract] OR "Ultrasonography"[Title/Abstract] OR "CT"[Title/Abstract] OR "computed tomography"[Title/Abstract] OR "Doppler"[Title/Abstract] OR "Echocardiography"[Title/Abstract] OR "Angiography"[Title/Abstract] OR "Angiogram"[Title/Abstract] OR "Arteriography"[Title/Abstract])

AND

("arteriosclerosis"[MeSH Terms] OR "plaque, atherosclerotic"[MeSH Terms] OR "carotid stenosis"[MeSH Terms] OR "carotid intima media thickness"[MeSH Terms] OR "coronary stenosis"[MeSH Terms] OR "vascular calcification"[MeSH Terms] OR "carotid artery diseases"[MeSH Terms] OR ("atherosclero*"[Title/Abstract] OR "arteriosclero*"[Title/Abstract] OR "plaque*"[Title/Abstract] OR "CIMT"[Title/Abstract] OR "intima media thickness"[Title/Abstract] OR "IMT"[Title/Abstract] OR "arterial wall thickness"[Title/Abstract] OR "coronary calc*"[Title/Abstract] OR "calcium score"[Title/Abstract] OR "vascular calcinosis"[Title/Abstract] OR "agatston score"[Title/Abstract] OR "steno*"[Title/Abstract] OR "occlus*"[Title/Abstract] OR "narrow*"[Title/Abstract] OR "atherogenesis"[Title/Abstract] OR "atheroma*"[Title/Abstract] OR "fibroatheroma*"[Title/Abstract]))

Embase

(exp preeclampsia/ OR pre eclamp*.ti,ab,kf. OR pre-eclamp*.ti,ab,kf. OR preeclamp*.ti,ab,kf. OR Toxemia.ti,ab,kf. OR Toxaemia.ti,ab,kf. OR eclamp*.ti,ab,kf. OR HELLP.ti,ab,kf. OR PE.ti,ab,kf. OR exp maternal hypertension/ OR exp HELLP syndrome/ OR maternal hypertension.ti,ab,kf.)

AND

(exp duplex Doppler ultrasonography/ OR exp echocardiography/ OR exp computed tomography angiography/ OR exp angiography/ OR exp diagnostic imaging/ OR Ultrasound.ti,ab,kf. OR Ultrasonography.ti,ab,kf. OR CT.ti,ab,kf. OR computed tomography.ti,ab,kf. OR Doppler.ti,ab,kf. OR Echocardiography.ti,ab,kf. OR Angiography.ti,ab,kf. OR Angiogram.ti,ab,kf. OR Arteriography.ti,ab,kf.)

AND

(exp arteriosclerosis/ OR exp atherosclerotic plaque/ OR exp carotid artery obstruction/ OR exp carotid intima-media thickness/ OR exp coronary artery obstruction/ OR exp blood vessel calcification/ OR exp carotid artery disease/ OR (atherosclero*.ti,ab,kf. OR arteriosclero*.ti,ab,kf. OR plaque*.ti,ab,kf. OR CIMT.ti,ab,kf. OR intima media thickness.ti,ab,kf. OR IMT.ti,ab,kf. OR arterial wall thickness.ti,ab,kf. OR coronary calc*.ti,ab,kf. OR calcium score.ti,ab,kf. OR vascular calcinosis.ti,ab,kf. OR agatston score.ti,ab,kf. OR steno*.ti,ab,kf. OR occlus*.ti,ab,kf. OR narrow*.ti,ab,kf. OR atherogenesis.ti,ab,kf. OR atheroma*.ti,ab,kf. OR fibroatheroma*.ti,ab,kf.))

Web of Science

(TS=("pre eclamp*" OR pre-eclamp* OR preeclamp* OR Toxemia OR Toxaemia OR eclamp* OR HELLP OR PE OR “maternal hypertension”)) AND (TS=(Arteriography OR angiogram OR angiography OR echocardiography OR doppler OR computed tomography OR CT OR ultrasound)) AND (TS=(fibroatheroma* OR atheroma* OR atherogenesis OR narrow* OR occlus* OR steno* OR "agatston score" OR "vascular calcinosis" OR “calcium score” OR "coronary calc*" OR "arterial wall thickness" OR IMT OR "intima media thickness" OR CIMT OR plaque* OR ateriosclero* OR atherosclero*))

**Table S1** Full-text articles excluded and reason for exclusion

| **Name of paper** | **Authors** | **Year** | **Exclusion reason** |
| --- | --- | --- | --- |
| Severity of obstructive coronary artery stenosis after pre-eclampsia. | Ray JG; Austin PC; Park AL; Cohen E; Fang J; Chu A | 2023 | Wrong study design |
| Impact of body mass index on markers of vascular health in normotensive women with history of pre-eclampsia. | Heidema WH; Van Drongelen J; Spaanderman MEA; Scholten RR | 2023 | Wrong outcome |
| Risk factor profiles of young women with vasomotor non-obstructive versus obstructive coronary syndromes: Importance of non-traditional and sex-specific risk factors | Theberge E.T.; Vikulova D.N.; Pimstone S.N.; Brunham L.R.; Humphries K.H.; Sedlak T.L. AO - Theberge, Emilie T. | 2023 | Wrong population |
| Structural and functional changes of the common carotid artery in patients with severe preeclampsia | Zhao W.; Cai Y.; Xu J. | 2023 | Wrong population |
| Long-term maternal cardiovascular risk after preeclampsia: a latent vascular enemy | Acevedo M.; Varleta P.; Tagle R. | 2023 | Wrong publication type |
| Increased Intima-Media Thickness of Carotid and Femoral Arteries in Women with Early Preeclampsia | Hassanzadeh, S; Livani, S; Azaderah, M; Madadi, M; Amlashi, FI | 2022 | Wrong population |
| Maternal Health: The Heart of the Matter | Fry E.T.A.; Wood M.J.; Walsh M.N. | 2022 | Wrong publication type |
| Evaluation of Cardiac Function of Pregnant Women with High Blood Pressure during Gestation Period and Coupling of Hearts with Peripheral Vessels by Ultrasonic Cardiogram under Artificial Intelligence Algorithm | Zhang X.; Liang X.; Cao W. | 2022 | Wrong population |
| Accelerated Coronary Atherosclerosis After Preeclampsia: Seeing Is Believing. | Honigberg MC; Jowell AR | 2022 | Wrong publication type |
| "Plaque erosion" or the danger of eerily quiet appearance | Caligiuri, G.; Mallat Z. | 2021 | Wrong publication type |
| Sex differences in the manifestation and evolution of coronary artery plaques | Bernhard, B; Grani, C | 2021 | Wrong publication type |
| Combined past preeclampsia and gestational diabetes is associated with a very high frequency of coronary microvascular dysfunction | S., Kul; T.S., Guvenc; O.F., Baycan; F.B., Celik; Z., Caliskan; R., Cetin Guvenc; F.C., Ciftci; M, Caliskan | 2021 | Wrong outcome |
| Sex, lies, and coronary artery disease | H., Martinez-Selles; D., Martinez-Selles; M, Martinez-Selles | 2021 | Wrong publication type |
| Postpartum microvascular functional alterations following severe preeclampsia | Barr, L C; Pudwell, J; Smith, G N | 2021 | Wrong outcome |
| Placental growth factor as a predictor of long-term cardiovascular risk | M.C., Adank; J.E.R., Van Lennep; L., Benschop; J.M., Roberts; R.E., Gandley; Y.B., De Rijke; E.A.P., Steegers; S, Schalekamp-Timmermans | 2021 | Wrong publication type |
| Increased prevalence of premature coronary atherosclerosis after preeclampsia | M.G., Hauge; P., Damm; K.F., Kofoed; A.S., Ersboell; M., Johansen; P.E., Sigvardsen; A., Fuchs; J.T., Kuhl; B.G., Nordestgaard; L., Koeber; F., Gustafsson; , Linde J J | 2021 | Wrong publication type |
| Dimethylarginines correlate to common carotid artery wall layer dimensions and cardiovascular risk factors in pregnant women with/without preeclampsia: A group comparative study. | Akhter, Tansim; Wikström, Gerhard; Larsson, Marita; Bondesson, Ulf; Hedeland, Mikael; Naessen, Tord | 2021 | Wrong outcome |
| Angiogenic factors and evaluation of vascular status in preeclampsia | S., Aksin; N., Cim; H.G., Sahin; Balsak D. | 2021 | Wrong outcome |
| Short term cardiovascular risk in normotensive women after hypertensive pregnancy | M.O., El Mokadem; Y.A.E., Hady; S.M., Yaquob | 2021 | Wrong population |
| Preeclampsia Is Associated With Increased Preclinical Carotid Atherosclerosis in Women With Type 1 Diabetes. | Amor, Antonio J; Vinagre, Irene; Valverde, Maite; Pané, Adriana; Urquizu, Xavier; Meler, Eva; López, Eva; Quirós, Carmen; Giménez, Marga; Codina, Laura; Conget, Ignacio; Barahona, Maria J; Perea, Verónica | 2020 | Reanalysis of same cohort |
| Cardiovascular health and vascular age after severe preeclampsia: A cohort study | Benschop, L; Schelling, S J C; Duvekot, J J; van Lennep, J E R | 2020 | Wrong study design |
| Adverse Pregnancy Outcomes Are Associated with Reduced Coronary Flow Reserve in Women With Signs and Symptoms of Ischemia Without Obstructive Coronary Artery Disease: A Report from the Women's Ischemia Syndrome Evaluation-Coronary Vascular Dysfunction Study. | Park, Ki; Quesada, Odayme; Cook-Wiens, Galen; Wei, Janet; Minissian, Margo; Handberg, Eileen M; Merz, Noel Bairey; Pepine, Carl J | 2020 | Wrong publication type |
| Comparative study of vascular function in normal and hypertensive pregnant | G., Chiarello; R., Carbonara; C., Girasoli; I., Panettieri; M., Ciccone; V, Vulpis | 2020 | Wrong publication type |
| Circulating neutrophils do not predict subclinical coronary artery disease in women with former preeclampsia | J.A.L., Meeuwsen; J., de Vries; G.A., Zoet; A., Franx; B.C.J.M., Fauser; A.H.E.M., Maas; B.K., Velthuis; Y.E., Appelman; F.L., Visseren; G., Pasterkamp; I.E., Hoefer; B.B., van Rijn; H.M., Den Ruijter; S.C.A., de Jager | 2020 | Wrong publication type |
| Platelet RNA modules point to coronary calcification in asymptomatic women with former preeclampsia. | Hartman, Robin J G; Korporaal, Suzanne J A; Mokry, Michal; de Jager, Saskia C A; Meeuwsen, John A L; van der Laan, Sander W; Lansu, Nico R; Zoet, Gerbrand A; Pasterkamp, Gerard; Urbanus, Rolf T; Hoefer, Imo E; Franx, Arie; Velthuis, Birgitta K; van Rijn, Bas B; den Ruijter, Hester M | 2019 | Wrong outcome |
| Vascular Outcomes of a Pregnancy Complicated by Preeclampsia | L.C., Barr; J., Pudwell; J.E., Herr; A., Johri; G.N., Smith | 2019 | Wrong publication type |
| Assessment of endothelial function in postpartum pre-eclamptic women using flow-mediated dilation of the brachial artery | P., Tripathy; A., Sahu; A., Nagy; J, Mohanty | 2019 | Wrong publication type |
| Association between reproductive factors and carotid atherosclerosis in post-menopausal women | A.-L., Madika; P., Nasserdine; S., Langlet; C., Lecerf; G., Ledieu; P., Devos; C, Mounier-Vehier | 2019 | Wrong publication type |
| Preeclampsia: A Risk Factor On Preclinical Carotid Atherosclerosis With Similar Impact To Type 1 Diabetes | A.J., Amor; I., Vinagre; M., Valverde; A., Pane; X., Urquizu; E., Meler; E., Lopez; C., Quiros; M., Gimenez; L., Codina; N., Alonso; I., Conget; M.J., Barahona; V, Perea | 2019 | Wrong publication type |
| The relationship between maternal characteristics and carotid intima-media thickness using an automated ultrasound technique | Santhirakumaran, S; Tay, J; Lees, C | 2019 | Wrong population |
| Sub-clinical atherosclerosis in the common carotid artery in women with/without previous pre-eclampsia: A seven-year follow-up | Akhter, Tansim; Larsson, Anders; Larsson, Marita; Naessen, Tord | 2019 | Wrong outcome |
| Pregnancy-related events associated with subclinical cardiovascular disease burden in late midlife: SWAN. | Cortés, Yamnia I; Catov, Janet M; Brooks, Maria; El Khoudary, Samar R; Thurston, Rebecca C; Matthews, Karen A; Isasi, Carmen R; Jackson, Elizabeth A; Barinas-Mitchell, Emma | 2019 | Wrong outcome |
| No long-term impairment of cerebral autoregulation after preeclampsia. | Janzarik, Wibke G; Gerber, Ann-Kathrin; Markfeld-Erol, Filiz; Sommerlade, Linda; Allignol, Arthur; Reinhard, Matthias | 2018 | Wrong outcome |
| Pregnancy history, coronary artery calcification and bone mineral density in menopausal women. | Beckman, J P; Camp, J J; Lahr, B D; Bailey, K R; Kearns, A E; Garovic, V D; Jayachandran, M; Miller, V M; Holmes, D R | 2018 | Not able to data extract |
| Prevalence of subclinical coronary artery disease assessed by coronary computed tomography angiography in 45- to 55-year- old women with a history of preeclampsia | Zoet, Gerbrand A.; Benschop, Laura; Boersma, Eric; Budde, Ricardo P.J.; Fauser, Bart C.J.M.; Van Der Graaf, Yolanda; De Groot, Christianne J.M.; Maas, Angela H.E.M.; Roeters Van Lennep, Jeanine E.; Steegers, Eric A.P.; Visseren, Frank L.; Van Rijn, Bas B.; Velthuis, Birgitta K.; Franx, Arie; Appelman, Yolande E.; Baart, Sara J.; Brouwers, Laura; Cannegieter, Suzanne C.; Dam, Veerle; Eijkemans, M. C.J.; Ferrari, Michel D.; Gunning, Marlise N.; Hoek, Annemieke; Koffijberg, Erik; Koster, M. P.H.; Kruit, Mark; Lagerwij, Giske R.; Lambalk, C. B.; Laven, Joop S.; Linstra, Katie; Van Der Lugt, Aad; Van Den Brink, Antoinette Maassen; Meun, Cindy; Middeldorp, Saskia; Moons, Karel G.M.; Roos-Hesselink, Jolien W.; Scheres, Luuk J.J.; Steegers-Theunissen, Regine P.M.; Terwindt, Gisela M.; Wermer, Marieke J.H. | 2018 | Reanalysis of same cohort |
| Association of pre-eclampsia with carotid artery intima-media thickness and non-alcoholic fatty liver disease | B., Memari; N., Moghiseh; F., Mohammadian; M., Ghajarzadeh; H, Ghoreishian | 2018 | Wrong population |
| History of preeclampsia and subclinical cardiovascular disease in mexican women | J., Mata; A., Catzin-Kulhmann; E., Ortiz-Panozo; C., Cantu-Brito; L., Espinosa; B., Rodriguez; M., Lajous; R, Lopez-Ridaura | 2018 | Wrong publication type |
| Cardiovascular RiskprofilE - IMaging and gender-specific disOrders (CREw-IMAGO): rationale and design of a multicenter cohort study. | Zoet, Gerbrand A; Meun, Cindy; Benschop, Laura; Boersma, Eric; Budde, Ricardo P J; Fauser, Bart C J M; de Groot, Christianne J M; van der Lugt, Aad; Maas, Angela H E M; Moons, Karl G M; Roeters van Lennep, Jeanine E; Roos-Hesselink, Jolien W; Steegers, Eric A P; van Rijn, Bas B; Laven, Joop S E; Franx, Arie; Velthuis, Birgitta K | 2017 | Wrong publication type |
| Gestational hypertension: Endothelial dysfunction as a marker of preeclampsia | R., Carbonara; F., Giardinelli; C., Carbonara; L., Zaccaro; R., Nitti; F., Clemente; I., Panettieri; A., Ciavarella; G., Loverro; M.M., Ciccone; V, Vulpis | 2017 | Wrong publication type |
| Reproductive factors associated with subclinical vascular measures at late midlife: Swan | Y.I., Cortes; J., Catov; M., Brooks; C., Isasi; E.A., Jackson; S., Harlow; K., Matthews; R., Thurston; , Barinas-Mitchell E | 2017 | Wrong publication type |
| Comparison of carotid artery intima media thickness in preeclamptic patients with healthy normotensive pregnant women | M., Afiat; E., Esmailpour; L., Jarahi; L., Pourali; G., Iranmanesh; N., Daghighi; P, Layegh | 2017 | Wrong outcome |
| Endothelial function and flow-mediated dilation of brachial artery in pre-eclampsia: color Doppler ultrasound study | Rezavand, N; Veisi, F; Zangeneh, M; Naghibi, S S; Seyedzadeh, S M S; Rezaei, M | 2017 | Wrong outcome |
| Vascular activation and development of carotid intima-media thickness in postmenopausal women is influenced by pregnancy history | V.M., Miller; V.D., Garovic; M.M., Mielke; K.R., Bailey; B., Lahr; W.M., White; M, Jayachandran | 2017 | Wrong publication type |
| Comparison of Abdominal Aorta Intima-Media Thickness, Serum Level of Leptin, and Lipid Profile between Maternal Preeclampsia and Neonatal Outcomes | Rezavand, N; Daryoushi, H; Veisi, F; Jalilian, N; Mehraban, N | 2017 | Wrong population |
| Serum Pentraxin 3 is associated with signs of arterial alteration in women with preeclampsia. | Akhter, Tansim; Wikström, Anna-Karin; Larsson, Marita; Larsson, Anders; Wikström, Gerhard; Naessen, Tord | 2017 | Wrong outcome |
| Association between angiogenic factors and signs of arterial aging in women with pre-eclampsia. | Akhter, T; Wikström, A-K; Larsson, M; Larsson, A; Wikström, G; Naessen, T | 2017 | Wrong outcome |
| Vascular ultrasound measures before pregnancy and pregnancy complications: A prospective cohort study. | Harville, Emily W; Juonala, Markus; Viikari, Jorma S A; Kähönen, Mika; Raitakari, Olli T | 2017 | Wrong outcome |
| Pregnancy complications and later vascular ultrasound measures: A cohort study | E.W., Harville; M., Juonala; J.S.A., Viikari; M., Kahonen; O.T., Raitakari | 2017 | Wrong outcome |
| Carotid Artery Intima-Media Thickness and Subclinical Atherosclerosis in Women With Remote Histories of Preeclampsia: Results From a Rochester Epidemiology Project-Based Study and Meta-analysis | V.D., Garovic; N.M., Milic; T.L., Weissgerber; M.M., Mielke; K.R., Bailey; B., Lahr; M., Jayachandran; W.M., White; H.N., Hodis; V.M., Miller | 2017 | Wrong outcome |
| Characterization of intravascular cellular activation in relationship to subclinical atherosclerosis in postmenopausal women | M., Jayachandran; V.D., Garovic; M.M., Mielke; K.R., Bailey; B.D., Lahr; V.M., Miller | 2017 | Wrong outcome |
| Pregnancy history and blood-borne microvesicles in middle aged women with and without coronary artery calcification | V.M., Miller; V.D., Garovic; K.R., Bailey; B.D., Lahr; M.M., Mielke; W.M., White; M, Jayachandran | 2016 | Reanalysis of same cohort |
| Sex-specific risk factors contributing to carotid intima-media thickness (CIMT) in menopausal women | V., Miller; M., Jayachandran; V.D., Garovic; M.M., Mielke; K.R., Bailey; B., Lahr; W.M., White | 2016 | Wrong outcome |
| Coronary artery calcification associates with specific cell-derived blood-borne microvesicles in middle-aged women with and without history of preeclampsia | M., Jayachandran; V.D., Garovic; B., Lahr; K.R., Bailey; M.M., Mielke; W.M., White; V.M, Miller | 2016 | Wrong publication type |
| A history of preeclampsia predicts coronary artery calcification three decades later | W.M., White; M.M., Mielke; B.D., Lahr; V.D., Miller; M., Jayachandran; W.A., Rocca; K.R., Bailey; V.D., Garovic | 2016 | Wrong publication type |
| Uterine Arcuate Artery Calcification on Transvaginal Sonography May Correlate With Known Risk Factors for Atherosclerosis | Ozdemir, M; Uzun, I; Ozel, A; Cakar, H; Inan, C; Yazicioglu, F | 2016 | Wrong population |
| Carotid artery intima-media thickness and resistance and pulsatility indices of uterine and renal arteries in pre-eclampsia: A quantitative analysis by color doppler ultrasonography | A., Ehsanbakhsh; N., Khorashadizadeh; M, Bohairaee | 2016 | Wrong population |
| Maternal subclinical vascular changes in fetal growth restriction with and without pre-eclampsia. | Stergiotou, I; Bijnens, B; Cruz-Lemini, M; Figueras, F; Gratacos, E; Crispi, F | 2015 | Wrong population |
| Vascular consequences of pre-eclampsia | C.E., Brown; J., Flynn; D.M., Carty; C, Delles | 2015 | Wrong publication type |
| Preeclampsia Is Associated with Increased Central Aortic Pressure, Elastic Arteries Stiffness and Wave Reflections, and Resting and Recruitable Endothelial Dysfunction | Torrado, J; Farro, I; Zocalo, Y; Farro, F; Sosa, C; Scasso, S; Alonso, J; Bia, D | 2015 | Wrong population |
| Persistence of cardiovascular risk factors in women with previous preeclampsia: A long-term follow-up study | Aykas, Fatma; Solak, Yalcin; Erden, Abdulsamet; Bulut, Kadir; Dogan, Selcuk; Sarli, Bahadr; Acmaz, Gokhan; Afsar, Baris; Siriopol, Dimitrie; Covic, Adrian; Sharma, Shailendra; Johnson, Richard J.; Kanbay, Mehmet | 2015 | Wrong outcome |
| Thicknesses of individual layers of artery wall indicate increased cardiovascular risk in severe pre-eclampsia. | Akhter, T; Larsson, M; Wikström, A K; Naessen, T | 2014 | Wrong outcome |
| Ultrasound study of carotid and cardiac remodeling and cardiac-arterial coupling in normal pregnancy and preeclampsia: a case control study. | Yuan, Li-Jun; Duan, Yun-You; Xue, Dan; Cao, Tie-Sheng; Zhou, Ning | 2014 | Wrong outcome |
| Follow up of intima-media thickness after severe early-onset preeclampsia | J., Blaauw; E.T.D., Souwer; S.M., Coffeng; A.J., Smit; J.J., Van Doormaal; M.M., Faas; M.G., Van Pampus | 2014 | Wrong outcome |
| Impaired coronary microvascular function and increased intima-media thickness in preeclampsia | F.C., Ciftci; M., Caliskan; O., Ciftci; H., Gullu; A., Uckuyu; E., Toprak; F, Yanik | 2014 | Wrong outcome |
| Does mild preeclampsia cause arterial stiffness and ventricular remodeling through inflammation? | Ciftci, Faika Ceylan; Ciftci, Ozgur; Gullu, Hakan; Caliskan, Mustafa; Uckuyu, Ayla; Ozcimen, Ebru Emel | 2014 | Wrong outcome |
| Myocardial performance index, aortic root diameter and carotid intima-media thickness in pregnancy-induced hypertension | G., Nasr; A., Nasr; A., Eleraki; S, Elrefai | 2014 | Wrong publication type |
| Preeclampsia in healthy women and endothelial dysfunction 10 years later. | Sandvik, Miriam Kristine; Leirgul, Elisabeth; Nygård, Ottar; Ueland, Per Magne; Berg, Ansgar; Svarstad, Einar; Vikse, Bjørn Egil | 2013 | Wrong outcome |
| Carotid arterial intima–media thickness and arterial stiffness in pre-eclampsia: analysis with a radiofrequency ultrasound technique. | Yuan, L J; Xue, D; Duan, Y Y; Cao, T S; Yang, H G; Zhou, N | 2013 | Wrong outcome |
| Individual common carotid artery wall layer dimensions, but not carotid intima-media thickness, indicate increased cardiovascular risk in women with preeclampsia: an investigation using noninvasive high-frequency ultrasound. | Akhter, Tansim; Wikström, Anna-Karin; Larsson, Marita; Naessen, Tord | 2013 | Wrong outcome |
| Vascular risk in women with a history of severe preeclampsia. | Goynumer, Gokhan; Yucel, Nese; Adali, Ertan; Tan, Temel; Baskent, Erdem; Karadag, Cihan | 2013 | Wrong outcome |
| Patterns of maternal vascular remodeling and responsiveness in early- versus late-onset preeclampsia. | Stergiotou, Iosifina; Crispi, Fatima; Valenzuela-Alcaraz, Brenda; Bijnens, Bart; Gratacos, Eduard | 2013 | Wrong population |
| Individual artery wall layer dimensions indicate increased cardiovascular risk in previous severe preeclampsia-an investigation using non-invasive high-frequency ultrasound | A., Tansim; L., Marita; W., Anna-Karin; , Tord N | 2013 | Wrong publication type |
| Unique features of long-term cardiovascular phenotype in young women with early-onset preeclampsia | M., Lazdam; A., De La Horra; J., Diesch; J., Francis; Y., Kenworthy; A., Shore; S., Neubauer; R., Kharbanda; N., Alp; C., Redman; B., Kelly; P, Leeson | 2012 | Wrong publication type |
| Carotid remodeling in preeclampsia by analysis with echo-tracking system | L.-J., Yuan; Y.-Y., Duan; D., Xue; H.-G., Yang; T.-S., Cao; N, Zhou | 2012 | Wrong publication type |
| Quantitative assessment of maternal common carotid artery mechanics using velocity vector imaging in pre-eclampsia | X.J., Ma; Y.Y., Duan; L.J., Yuan; T.S., Cao; Y., Wang; H.G., Yang; S., Chen | 2012 | Wrong population |
| Vascular adaptations to 12-weeks cycling training in formerly preeclamptic women | R.R., Scholten; D., Thijssen; F.K., Lotgering; M.T.E., Hopman; M.E.A., Spaanderman | 2012 | Wrong publication type |
| Long-term cardiac and vascular phenotype of young women with pregnancies complicated by preeclampsia | M., Lazdam; A., De La Horra; J., Diesch; J., Francis; Y., Kenworthy; A., Shore; C., Redman; S., Neubauer; R., Kharbanda; N., Alp; B., Kelly; P, Leeson | 2012 | Wrong publication type |
| Peripheral arterial tonometry (PAT) technology to assess endothelial function in pregnancy | C., Delles; D.M., Carty; L.A., Anderson; C., Nicolson Duncan; D.P., Baird; L., Rooney; A.F., Dominiczak | 2011 | Wrong publication type |
| Structural vascular changes in young women with previous preeclampsia: Link to cardiovascular disease development? | M., Lazdam; A., De La Horra; J., Diesch; C., Szmigielski; R.K., Kharbanda; N., Alp; B., Kelly; P., Leeson | 2011 | Wrong publication type |
| Intima-media thickness and hypertensive disorders of pregnancy: a prospective study | Verissimo, Carlos | 2010 | Wrong outcome |
| Longitudinal study on cardiovascular risk markers in women with a history of severe early-onset preeclampsia | J., Blaauw; E.T.D., Souwer; S., Coffeng; A.J., Smit; J.G., Aarnoudse; J.J., Van Doormaal; M.M., Faas; M.G., Van Pampus | 2010 | Wrong publication type |
| Risk factors for premature coronary disease in women | Saldarriaga, C I; Franco, G; Garzon, A M; Garcia, I; Mejia, N; Restrepo, A | 2010 | Wrong outcome |
| Hypertension during Pregnancy is Associated with Coronary Artery Calcium Independent of Renal Function | Cassidy-Bushrow, A E; Bielak, L F; Rule, A D; Sheedy, P F; Turner, S T; Garovic, V D; Peyser, P A | 2009 | Wrong population |
| Expression of inflammatory factors and increasing of the intima-media thickness in pre-eclampsia: evidence of maternal and neonatal arteriosclerotic risk | J.E., Valdivia-Silva; A., Cardenas; S, Medina | 2009 | Wrong population |
| Association of remote hypertension in pregnancy with coronary artery disease a case-control study | G., Valdes; F., Quezada; E., Marchant; A., Von Schultzendorff; S., Moran; O., Padilla; A, Martinez | 2009 | Wrong population |
| Subclinical atherosclerosis in association with elevated placental vascular resistance in early pregnancy | Lausman, A Y; Kingdom, J C; Bradley, T J; Slorach, C; Ray, J G | 2009 | Wrong population |
| High blood pressure in pregnancy and coronary calcification | Sabour, S; Franx, A; Rutten, A; Grobbee, D E; Prokop, M; Bartelink, M L; van der Schouw, Y T; Bots, M L | 2007 | Wrong population |
| Increased intima-media thickness after early-onset preeclampsia | J., Blaauw; M.G., Van Pampus; J.J., Van Doormaal; M.R., Fokkema; V., Fidler; A.J., Smit; J.G., Aarnoudse | 2006 | Wrong outcome |
| Vascular reactivity in preeclampsia assessed noninvasively using maternal brachial artery hyperemic response. | Williams, Keith; Kocer, Cenk | 2004 | Wrong population |
| Risk for subsequent coronary artery disease after preeclampsia. | Haukkamaa, Leena; Salminen, Minna; Laivuori, Hannele; Leinonen, Hannu; Hiilesmaa, Vilho; Kaaja, Risto | 2004 | Wrong population |
| Ultrasonography in the diagnosis of cerebrovascular disorders in pregnancy complicated by gestosis | Muratov, F Kh; Kravtsova, E M | 2001 | Wrong population |

**Table S2** Complete definitions of pre-eclampsia used by included studies

| **Pre-eclampsia criteria** | **Definition** |
| --- | --- |
| American College of Obstetrics & Gynecology (ACOG) 2020^1^ | High blood pressure (Systolic blood pressure of 140 mm Hg or more or diastolic blood pressure of 90 mm Hg or more on two occasions at least 4 hours apart after 20 weeks of gestation in a woman with a previously normal blood pressure)  AND  Proteinuria (300 mg or more per 24 hour urine collection, or Protein/creatinine ratio of 0.3 mg/dL or Dipstick reading of 2+  Or in absence of proteinuria, new onset hypertension with new onset of any of the following:   - Thrombocytopenia: Platelet count less than 100,000 × 10^9^/L - Renal insufficiency: Serum creatinine concentrations greater than 1.1 mg/dL or a doubling of the serum creatinine concentration in the absence of other renal disease - Impaired liver function: Elevated blood concentrations of liver transaminases to twice normal concentration - Pulmonary edema - New-onset headache unresponsive to medication and not accounted for by alternative diagnoses or visual symptoms |
| International Society for the Study of Hypertension in Pregnancy (ISSHP) 2001^2^ | De novo hypertension (≥140 mm Hg systolic and/or ≥90 mm Hg diastolic) after 20 weeks of gestation and proteinuria (≥300 mg/24 h or >1+ in the dipstick test) |
| International Society for the Study of Hypertension in Pregnancy (ISSHP) 2014^3^ | De novo hypertension (≥140 mm Hg systolic and/or ≥90 mm Hg diastolic) after 20 weeks of gestation  AND  Proteinuria (Spot urine protein/creatinine ≥30mg/mol or ≥300 mg/24 h or >1+ in the dipstick test)  OR  Maternal organ dysfunction:   - Renal insufficiency (creatinine ≥90umol/L; 1.02mg/dL) - Liver involvement (elevated transaminases – at least twice upper limit of normal +- right upper quadrant or epigastric abdominal pain) - Neurological complications (e.g. eclampsia, altered mental status, blindness, stroke, or more commonly hyperreflexia when accompanied by clonus, severe headaches when accompanied by hyperreflexia, persistent visual scotomata) - Haematological complications (thrombocytopenia <150,000/dL, DIC, haemolysis)   OR  Uteroplacental dysfunction leading to foetal growth restriction |
| International Statistical Classification of Diseases and Related Health Problems 7^th^ Revision (ICD-7)^4^ | 642 – Toxaemias of pregnancy |
| International Statistical Classification of Diseases and Related Health Problems 8^th^ Revision (ICD-8)^5^ | 637 – Pre-eclampsia, eclampsia and toxaemia, unspecified  637.03 – Pre-eclampsia mild  637.04 – Pre-eclampsia severe  637.09 – Pre-eclampsia unspecified  637.10 – Eclampsia  637.99 - Toxicosis  643.10 – Spontaneous abortion with toxemia  644.10 – Abortion not specified as induced or spontaneous with toxemia |
| International Statistical Classification of Diseases and Related Health Problems 9^th^ Revision (ICD-9)^6^ | 642E – Mild or unspecified pre-eclampsia  642F – Severe pre-eclampsia  642G – Eclampsia unspecified  642H – Pre-eclampsia or eclampsia superimposed on pre-existing hypertension  6424-6427 – Pre-eclampsia or eclampsia  6461A – Edema or excessive weight gain pregnancy, without mention of hypertension  6462A – Unspecified renal disease without mention of hypertension |
| International Statistical Classification of Diseases and Related Health Problems 10^th^ Revision (ICD-10)^7^ | O11 – Pre-eclampsia superimposed on chronic hypertension  O14 – Pre-eclampsia  O14.0 – Pre-eclampsia mild/moderate  O14.1 – Pre-eclampsia severe  O14.2 – HELLP syndrome  O14.9 – Pre-eclampsia unspecified  O15 - Eclampsia  O15.0 – Eclampsia in pregnancy  O15.9 – Eclampsia, unspecified as to time period  Z358Q – History of severe pre-eclampsia/HELLP |
| National High Blood Pressure Education Program Working group 1990^8^ | Onset of hypertension (blood pressure ≥140/90 mmHg) after 20  weeks gestation, accompanied by proteinuria (>300 mg per 24 h  urine collection or ≥2+ protein on urine dipstick). |
| Validated preeclampsia questionnaire^9^ | Questionnaire questions:   1. Are you currently taking prescription medication to lower your blood pressure?    - a) Yes    - b) No    - c) Do not know 2. Have you had at least one pregnancy that lasted more than 6 mo?    - a) Yes    - b) No 3. During any of these pregnancies (which lasted more than 6 mo), did a physician ever tell you that you had high blood pressure or hypertension?    - a) Yes      - a In the first pregnancy only      - b Not in the first, but in a subsequent pregnancy      - c In the first pregnancy and in at least 1 subsequent pregnancy    - b) No      - a During any of your pregnancies, did you have preeclampsia, eclampsia, or toxemia?        - I Yes        - Ii No   4. In what year, or how old were you, when the pregnancy-related high blood pressure first occurred?  a. Year____ or Age __   1. During any of the pregnancies in which you developed hypertension, did you have:    - a) Protein in the urine    - b) Seizures or convulsions    - c) Preeclampsia, eclampsia, or toxemia of pregnancy 2. Before the first pregnancy in which you developed hypertension, did you have:    - a) Protein in the urine    - b) Seizures or convulsions    - c) High blood pressure (hypertension)   Questionnaire interpretation:  A positive response (ie, verification of preeclampsia) was defined as a self-report of any of the following:   - 1 History of preeclampsia, eclampsia, or toxemia, with or without hypertension, during the index pregnancy. - 2 Hypertension and proteinuria during, but not before the index pregnancy. - 3 Hypertension and seizures during, but not before the index pregnancy. |

**Table S3** Quality assessment of included studies using Newcastle–Ottawa scale

| **First Author; year** | **Selection** | | | | **Comparability** | **Outcome** | | | **Overall** | |
| --- | --- | --- | --- | --- | --- | --- | --- | --- | --- | --- |
|  | Representativeness of exposed cohort | Selection of non-exposed cohort | Ascertainment of exposure | Demonstration that outcome of interest was not present at start of study | Comparability of cohorts on the basis of the design or analysis controlled for confounders | Assessment of outcome | Was follow-up long enough for outcomes to occur | Adequacy of follow-up of cohorts | Total score | AHRQ quality standard* |
| Age group 30-39 | | | | | | | | | | |
| Barr *et al.* (2022) | / | / | / | / | ** | * | * | / | 4 | Poor |
| Age group 40-49 | | | | | | | | | | |
| Amor *et al. (*2021) | * | * | * | / | ** | * | * | / | 7 | Good |
| Benschop *et al. (*2020) | * | / | * | / | ** | * | * | / | 6 | Fair |
| Christensen et al. (2016) | * | * | * | / | * | * | * | / | 6 | Good |
| Hauge *et al.* (2022) | * | / | * | / | ** | * | * | / | 6 | Fair |
| McDonald *et al. (*2013) | * | / | * | / | ** | * | * | / | 6 | Fair |
| Wichmann *et al.* (2019) | * | * | * | / | ** | * | * | / | 7 | Good |
| Age group 50-60 | | | | | | | | | | |
| Al-Gburi *et al.* (2022) | * | * | / | / | ** | * | * | / | 6 | Fair |
| Haukkamaa *et al.*  (2009) | * | * | * | / | ** | * | * | / | 7 | Good |
| Lawesson *et al.* (2023) | * | * | * | / | ** | * | * | / | 7 | Good |
| White *et al.* (2016) | * | * | * | / | ** | * | * | / | 7 | Good |
| **Average score** | **2.4** | | | | **1.9** | **2** | | | **6.2** | |

AHRQ study quality standard: poor - selection 0-1 star or comparability 0 stars or outcome / exposure 0-1 star; fair - selection 2 stars and comparability 1-2 stars and outcome / exposure 2-3 stars; good - selection 3-4 stars and comparability 1-2 stars and outcome / exposure 2-3 stars

**Table S4** Odds ratios for prevalence of atherosclerotic plaque after pre-eclamptic pregnancy (cases) vs non-pre-eclamptic pregnancy (controls), according to type of plaque and maternal age at evaluation

| **First Author; year** | **Any plaque:** | **Cases (n) / Controls (n)** | **Cases:**  **Plaques n (%)** | **Controls:**  **Plaques n (%)** | **Odds ratio**  **(95% Confidence interval);**  **p-value** | **Calcified plaque:** | **Cases (n) / Controls (n)** | **Cases:**  **Plaques n (%)** | **Controls:**  **Plaques n (%)** | **Odds ratio**  **(95% Confidence interval);**  **p-value** | **Non-calcified plaque:** | **Cases (n) / Controls (n)** | **Cases:**  **Plaques n (%)** | **Controls:**  **Plaques n (%)** | **Odds ratio**  **(95% Confidence interval);**  **p-value** |
| --- | --- | --- | --- | --- | --- | --- | --- | --- | --- | --- | --- | --- | --- | --- | --- |
| Age group 30-39 | | | | | | | | | | | | | | | |
| Barr *et al.* (2022)^21^ | Mannheim plaque consensus - focal structure protruding into the arterial lumen by at least 50% of the surrounding intima-media thickness or measuring >=1.5mm in thickness from the vessel wall.†^2^ | 30/30 | 2 (6.7) | 3 (10.0) | 0.64 (0.10-4.15);  0.64 |  |  |  |  |  |  |  |  |  |  |
| Age group 40-49 | | | | | | | | | | | | | | | |
| Amor *et al.* (2021)^20^ | Focal wall thickenings encroaching into the arterial lumen by at least 50% of the surrounding IMT value or with a thickness of at least 1.5mm, as measured from the media adventitia interphase to the intima-lumen surface.†^2^ | 28/28 | 7 (25.0) | 2 (7.1) | 4.33 (0.81-23.10);  0.09 |  |  |  |  |  |  |  |  |  |  |
| Benschop *et al.* (2020)^22^ | *Calcified, non-calcified, mixed plaque.^¶^* | *258/** | *88 (34.1)* | *** | *** | CAC>0†^2^,‡^2^ | 258/644 | 58 (22.5) | 88 (13.7) | 1.83 (1.27-2.65)  <0.01 |  |  |  |  |  |
|  |  |  |  |  |  | CAC>10 | 258/644 | 47 (18.2) | 54 (8.4) | 2.43 (1.60-3.71);  <0.01 |  |  |  |  |  |
|  |  |  |  |  |  | CAC>100 | 258/644 | 14 (5.4) | 17 (2.6) | 2.12 (1.03-4.36);  0.04 |  |  |  |  |  |
| Christensen *et al.* (2016)^23^ | Plaque - Focal increase in cIMT above 1.5mm in both longitudinal and cross-sectional views.†^2^ | 20/20 | 2 (10.0) | 1 (5.0) | 2.11 (0.18-25.35);  0.56 |  |  |  |  |  |  |  |  |  |  |
| Hauge *et al.* (2022)^24^ | Atherosclerosis <50% and >=50% of arterial lumen or CAC>0.†^2^ | 704/706 | 193 (27.4) | 141 (20.0) | 1.51 (1.18-1.94);  <0.01 | Any calcified plaque.‡^2^ | 704/706 | 117 (16.6) | 83 (11.8) | 1.50 (1.10-2.03);  0.01 | Non-calcified plaque only - any coronary plaque on coronary CTA based on coronary artery luminal smoothness and overall calibre (SCCT guidelines). §^2^ | 704/706 | 76 (10.8) | 58 (8.2) | 1.35 (0.94-1.94);  0.10 |
|  |  |  |  |  |  | CAC>0 | 704/706 | 117 (16.6) | 83 (11.8) | 1.50 (1.10-2.03);  0.01 |  |  |  |  |  |
| McDonald *et al.* (2013)^26^ | Discrete >=1mm protrusions into the lumen in the common carotid, bifurcation, and proximal internal carotid arteries. Any plaque counted if at least one segment had plaque (or calcifications). †^2^ | 109/219 | 39 (35.8) | 55 (25.1) | 1.66 (1.01-2.73);  0.05 | Any calcified plaque.‡^2^ | 109/219 | 16 (14.7) | 25 (11.4) | 1.34 (0.68-2.62);  0.40 | Any non-calcified plaque.§^2^ | 109/219 | 23 (21.1) | 30 (13.7) | 1.68 (0.92-3.07);  0.09 |
| Wichmann *et al.* (2019)^29^ | Visible calcified, non-calcified, mixed plaque (>=20% luminal narrowing).†^2^ | 137/445 | 41 (29.9) | 106 (23.8) | 1.37 (0.89-2.09);  0.15 |  |  |  |  |  |  |  |  |  |  |
| Age group 50-60 | | | | | | | | | | | | | | | |
| Al-Gburi *et al.* (2022)^19^ |  |  |  |  |  | CAC>0†^2^,‡^2^ | 100/100 | 22 (22.0) | 11 (11.0) | 2.28 (1.04-5.00);  0.04 |  |  |  |  |  |
| Haukkamaa *et al.*  (2009)^25^ | Focal raised lesion of >1.5mm in size in at least 1 image of the carotid bulb.†^2^ | 35/489 | 10 (28.6) | 59 (12.1) | 3.00 (1.37-6.53);  0.01 |  |  |  |  |  |  |  |  |  |  |
| Lawesson *et al.* (2023)^27^ | Any coronary atherosclerosis.†^2^ | 499/8537 | 181 (36.3) | 2414 (28.3) | 1.44 (1.20-1.74);  <0.01 | Any calcified plaque.‡^2^ | 499/8537 | 136 (27.3) | 1999 (23.4) | 1.23 (1.00-1.50);  0.05 | Any non-calcified plaque.§^2^ | 499/8537 | 45 (9.0) | 415 (4.9) | 1.94 (1.41-2.68);  <0.01 |
|  |  |  |  |  |  | CAC>100 | 496/8473 | 45 (9.1) | 421 (5.0) | 1.91 (1.38-2.63);  <0.01 |  |  |  |  |  |
| White *et al.* (2016)^28^ |  |  |  |  |  | CAC>0†^2^,‡^2^ | 39/40 | 19 (48.7) | 10 (25.0) | 2.85 (1.10-7.38);  0.03 |  |  |  |  |  |
| ***Age group 30-39*** | **Any plaque** | ***30/30*** | ***2 (6.7)*** | ***3 (10.0)*** | ***0.64 (0.10-4.15);***  ***0.64*** | **Any calcified plaque** | ******* | ******* | ******* | ******* | **Any non-calcified plaque** | ******* | ******* | ******* | ******* |
| ***Age group 40-49*** | **Any plaque** | ***1256/2062*** | ***340 (27.1)*** | ***393 (19.1)*** | ***1.59 (1.34-1.89);***  ***<0.01*** | **Any calcified plaque** | ***1071/1569*** | ***191 (17.8)*** | ***196 (12.4)*** | ***1.59 (1.27-1.98);***  ***<0.01*** | **Any non-calcified plaque** | ***813/925*** | ***99 (12.2)*** | ***88 (9.5)*** | ***1.43 (1.05-1.95);***  ***0.02*** |
| ***Age group 50-60*** | **Any plaque** | ***673/9166*** | ***232 (34.5)*** | ***2494 (27.2)*** | ***2.00 (1.30-3.08);***  ***<0.01*** | **Any calcified plaque** | ***638/8677*** | ***177 (27.7)*** | ***2020 (23.3)*** | ***1.73 (0.99-3.01);***  ***0.05*** | **Any non-calcified plaque** | ***499/8537*** | ***45 (9.0)*** | ***415 (4.9)*** | ***1.94 (1.41-2.68);***  ***<0.01*** |
| **Pooled overall result** | **Any plaque†^1^** | **1959/11,258** | **574 (29.3)** | **2890 (25.7)** | **1.57 (1.39-1.78);**  **<0.01** | **Any calcified plaque‡^1^** | **1709/10,246** | **368 (21.5)** | **2216 (21.6)** | **1.52 (1.23-1.88);**  **<0.01** | **Any non-calcified plaque§^1^** | **1312/9462** | **144 (11.0)** | **503 (5.3)** | **1.65 (1.27-2.14);**  **<0.01** |

*Information/specific data not available, †^1^For ‘any plaque’, in absence of collated any plaque data, calcified plaque data was used indicated by †^2^. †^2^Study variable used to determine pooled overall results for ‘any plaque’. ‡^1^For ‘Any calcified plaque’, the variable indicated by ‡^2^ was used. ‡^2^Study variable used to determine pooled overall results for ‘any calcified plaque’. §^1^For non-calcified plaque, the variable indicated by §^2^ was used. §^2^Study variable used to determine pooled overall results for ‘any non-calcified plaque’. ¶Contrast CT only carried out in case group.

**Figure S1** Forest plot showing sensitivity analysis for presence of any atherosclerotic plaque after pre-eclamptic pregnancy (cases) *vs* non-pre-eclamptic pregnancy (controls), stratified by average maternal age at evaluation. Only studies in which odds ratios were adjusted for confounders are included.


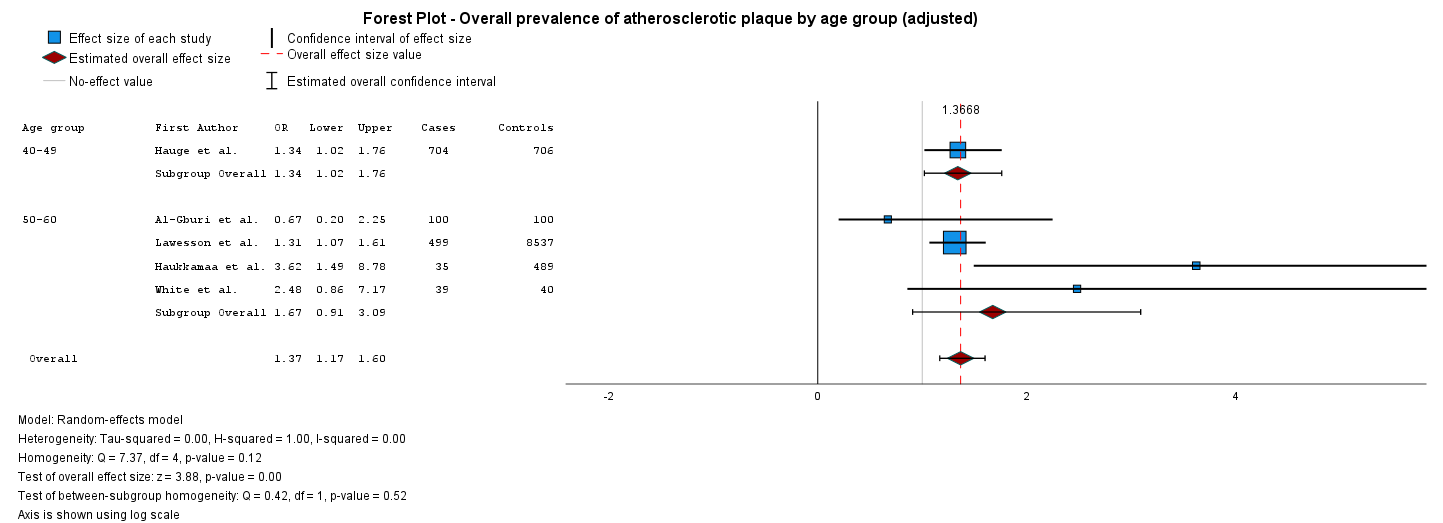


Five articles included with adjusted odds ratios. Variables adjusted for: Hauge - Age, parity, dyslipidaemia, diabetes, smoking, BMI, menopause, hypertension; Al-Gburi - Age, waist circumference, BMI, DBP, glucose, hypertension; Lawesson - Age, smoking, diabetes, SBP, hypertension, statin use, BMI, HDL cholesterol, total cholesterol; Haukkamaa - Age, hs-CRP, glucose, age at menarche, SBP, BMI, Parity, insulin, smoking, HDL cholesterol; and White - BMI, hypertension.

**Figure S2** Forest plot showing sensitivity analysis for presence of any atherosclerotic plaque after pre-eclamptic pregnancy (cases) *vs* non-pre-eclamptic pregnancy (controls), stratified by imaging modality used/artery visualized. Only studies in which odds ratios were adjusted for confounders are included.

***
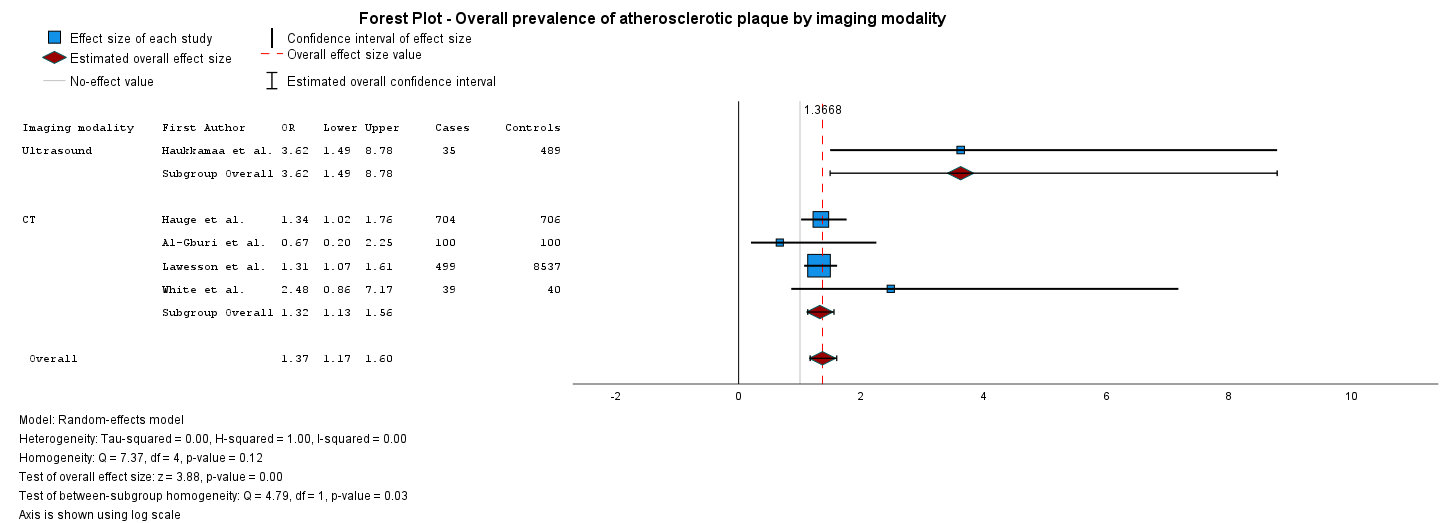
***

Five articles included with adjusted odds ratios. Variables adjusted for: Hauge - Age, parity, dyslipidaemia, diabetes, smoking, BMI, menopause, hypertension; Al-Gburi - Age, waist circumference, BMI, DBP, glucose, hypertension; Lawesson - Age, smoking, diabetes, SBP, hypertension, statin use, BMI, HDL cholesterol, total cholesterol; Haukkamaa - Age, hs-CRP, glucose, age at menarche, SBP, BMI, Parity, insulin, smoking, HDL cholesterol; and White - BMI, hypertension.

**Figure S3** Meta-regression bubble plot adjusted odds ratio for prevalence of atherosclerotic plaque after pre-eclamptic *vs* non-pre-eclamptic pregnancy, moderated by average maternal age at evaluation. Only studies in which odds ratios were adjusted for confounders are included. Red line shows where odds ratio becomes significant.

*
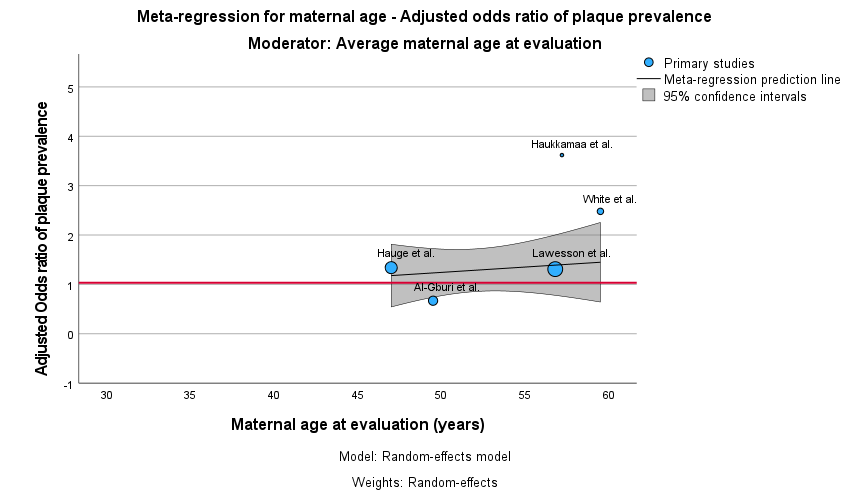
*

**Figure S4** Funnel plot showing distribution of studies included in meta-analysis according to odds ratio for prevalence of atherosclerotic plaque after pre-eclamptic *vs* non-pre-eclamptic pregnancy.

**
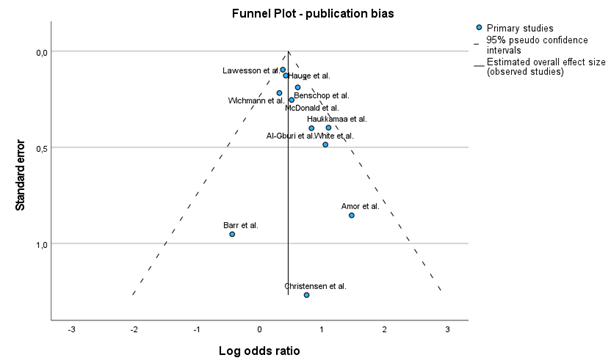
**
